# MARCH8 Restricts RSV Replication by Promoting Cellular Apoptosis Through Ubiquitin-Mediated Proteolysis of Viral SH Protein

**DOI:** 10.3390/v16121935

**Published:** 2024-12-18

**Authors:** Takashi Okura, Tatsuki Takahashi, Taichi Kameya, Fuminori Mizukoshi, Yusuke Nakai, Masatoshi Kakizaki, Mayuko Nishi, Noriyuki Otsuki, Hirokazu Kimura, Kei Miyakawa, Kazuya Shirato, Wataru Kamitani, Akihide Ryo

**Affiliations:** 1 Department of Virology 3, National Institute of Infectious Diseases, Musashimurayama 208-0011, Tokyo, Japan; t-okura@niid.go.jp (T.O.); t-kameya@niid.go.jp (T.K.); mzksh@niid.go.jp (F.M.); yusuke06@niid.go.jp (Y.N.); kakizaki@niid.go.jp (M.K.); mnishi@niid.go.jp (M.N.); otsuki@niid.go.jp (N.O.); shirato@niid.go.jp (K.S.); 2 Department of Infectious Diseases and Host Defense, Graduate School of Medicine, Gunma University, Maebashi 371-8511, Gunma, Japan; ta-takahashi@gunma-u.ac.jp (T.T.); wakamita@gunma-u.ac.jp (W.K.); 3 Life Science Laboratory, Technology and Development Division, Kanto Chemical Co., Inc., Chuo-ku 259-1146, Kanagawa, Japan; 4 Department of Health Science, Graduate School of Health Sciences, Gunma Paz University, Takasaki 370-0006, Gunma, Japan; h-kimura@paz.ac.jp; 5 Research Center for Influenza and Respiratory Viruses, National Institute of Infectious Diseases, Musashimurayama 208-0011, Tokyo, Japan; keim@niid.go.jp; 6 Department of Microbiology, Graduate School of Medicine, Yokohama City University, Yokohama 236-0004, Kanagawa, Japan

**Keywords:** respiratory syncytial virus, small hydrophobic protein, MARCH8, ubiquitination, apoptosis

## Abstract

Numerous host factors function as intrinsic antiviral effectors to attenuate viral replication. MARCH8 is an E3 ubiquitin ligase that has been identified as a host restriction factor that inhibits the replication of various viruses. This study elucidated the mechanism by which MARCH8 restricts respiratory syncytial virus (RSV) replication through selective degradation of the viral small hydrophobic (SH) protein. We demonstrated that MARCH8 directly interacts with RSV-SH and catalyzes its ubiquitination at lysine 13, leading to SH degradation via the ubiquitin-lysosomal pathway. Functionally, MARCH8 expression enhances RSV-induced apoptosis through SH degradation, ultimately reducing viral titers. Conversely, an RSV strain harboring the SH-K13R mutation exhibited prolonged SH protein stability and attenuated apoptosis in infected cells, even in the presence of MARCH8. Targeted depletion of MARCH8 enhances cellular survival and potentially increases viral persistence. These findings demonstrate that MARCH8 promotes the early elimination of virus-infected cells by abrogating the anti-apoptotic function of SH, thereby reducing viral transmission. Our study provides novel insights into the interplay between host restriction factors and viral evasion strategies, potentially providing new therapeutic approaches for RSV infections.

## 1. Introduction

Respiratory syncytial virus (RSV) is a significant pathogen that primarily infects infants and young children and causes acute respiratory infections characterized by symptoms of bronchitis and pneumonia. Premature infants and those with underlying medical conditions are at a particularly high risk of severe complications, with an estimated 100,000–200,000 deaths reported annually [1]. In recent years, RSV has emerged as a serious threat to even adults and elderly with chronic respiratory diseases, with mortality rates comparable to those of seasonal influenza viruses [2]. Currently, two RSV vaccines are available: GSK Arexvy and Pfizer Abrysvo, which are used in elderly and pregnant women [3]. Additionally, humanized neutralizing antibodies, such as nilcevimab and palivizumab, are employed for prophylactic use in high-risk infants. However, these antibodies are expensive and require multiple administrations [4,5]. Despite the availability of RSV vaccines and prophylactic antibodies, the establishment of multiple treatment and prevention strategies to effectively control this highly pathogenic and infectious virus is crucial.

RSV belongs to the genus Orthopneumovirus within the family Pneumoviridae and possesses a non-segmented negative-sense single-stranded RNA genome of approximately 15 kbp [6]. The viral genome encodes 11 viral proteins, including viral membrane glycoproteins (G), fusion proteins (F), and small hydrophobic proteins (SH) [7]. RSV is classified into subgroups A and B based on their G gene sequence [8]. Of these, the G, F, and SH proteins are present as membrane proteins on the surface of infectious virus particles. The SH protein is a pentameric glycoprotein comprising approximately 64 amino acids that exhibit pH-dependent ion channel activity and function as a viroporin [9], although the precise role in viral replication remains unclear. Several studies have suggested that SH protein is involved in viral virulence, as SH-deficient viruses have demonstrated attenuated severity in animal models [10]. Another crucial function of the SH protein is the inhibition of apoptosis through inhibiting TNF-alpha signaling, leading to the attenuation of caspase-8 activity [11,12]. Cell death plays a vital role in the eradication of infected viruses, suppression of viral proliferation, and prevention of viral spread to the surrounding non-infected cells [13]. However, it remains to be elucidated how SH-mediated inhibition of apoptosis affects viral proliferation and whether host factors counteract this function.

MARCH8 is a member of the Membrane-Associated RING-CH (MARCH) family of E3 ubiquitin ligases and has emerged as a significant player in innate antiviral immunity. Some viral membrane proteins are ubiquitinated by MARCH8, and the ubiquitinated viral proteins are endocytosed from the cell membrane and subsequently degraded in lysosomes. Therefore, this mechanism can serve as a host defense strategy, reducing the presence of viral components in infected cells and potentially limiting viral spread. [14]. Several studies unveiled MARCH8′s potent antiviral properties, particularly against enveloped viruses [15,16]. The antiviral activity of MARCH8 was first prominently demonstrated against HIV-1 [17]. MARCH8 also exhibits inhibitory effects against other enveloped viruses, including influenza A virus (IAV), Ebola virus (EBOV), and vesicular stomatitis virus (VSV) [17,18,19]. The broad-spectrum antiviral activity of MARCH8 suggests a conserved mechanism that potentially targets the common features of enveloped viruses. However, there are no reports that MARCH8 suppresses RSV, a clinically important respiratory enveloped virus.

In this study, we report that MARCH8 specifically targets the RSV-SH protein for ubiquitination and promotes its degradation through the ubiquitin-lysosomal pathway. We propose that SH degradation serves as a host defense mechanism to efficiently eliminate infected cells by attenuating the apoptosis-inhibitory activity of SH. Furthermore, we identified a novel SH mutant capable of evading suppression by MARCH8, revealing a previously unknown virus–host antagonism. These findings provide important insights that may contribute to the development of novel therapeutic strategies against RSV infections.

## 2. Materials and Methods

### 2.1. Cells

293T (ATCC: CRL-11268), A549 (ATCC: CCL-185), and HEp-2 (ATCC: CCL-23) cells were maintained in Dulbecco’s modified Eagle’s medium (DMEM) supplemented with 10% fetal bovine serum (FBS) and 1% penicillin-streptomycin. To generate MARCH8-knockdown A549 cells, the cells were transduced with lentiviral particles carrying gene-specific shRNAs against MARCH8 (Sigma-Aldrich, St. Louis, MO, USA, cat. no. TRCN0000073234, and TRCN0000073237) and then selected using 1.5 mg/mL puromycin (InvivoGen, San Diego, CA, USA, Cat. No. ant-pr-1).

### 2.2. Plasmids

The hemagglutinin (HA) tag and mStrawberry gene were added to the SH gene from the RSV/A/NIID/2370/14 strain (GenBank: LC474558) at the C-terminus and cloned into pCAGGS. An SH mutant with a lysine-to-arginine substitution at amino acid position 13 in CT (SH-K13R) was generated using overlapping PCR. The M2 gene of the influenza A/Puerto Rico/8/34 (PR8) virus fused with mStrawberry at the C-terminus was cloned into pCAGGS. The MARCH1, 2, 4, 6, and 8 constructs were purchased from Promega, Madison, WI, USA (Cat. No. FHC02473, FHC26444, FHC00826, FHC01100, and FHC11547, respectively), and MARCH8 and MARCH8-HA as cloned into pCAGGS. The MARCH8-W114A mutant construct was similarly generated by overlapping PCR. The ubiquitin gene was tagged with a FLAG tag, and the human Rab7 gene fused to AcGFP was cloned into pCAGGS.

### 2.3. BAC Constructions and Recovery for Recombinant RSV by Reverse Genetics

A bacterial artificial chromosomal (BAC) DNA clone of pBAC-T7-rA2-ZsGreen was used as the backbone [20], referred to as the wild-type (wt) strain in the present study. The BAC clone for the SH mutant with a lysine-to-arginine substitution at amino acid position 13 (SH-K13R) was generated using a Red/ET Recombination System Counter-Selection BAC Modification Kit according to the manufacturer’s instructions. Wild-type and mutant SH-K13R viruses were generated by a reverse genetics system using BAC DNAs and support plasmids as previously described [20]. Briefly, 293T cells were co-transfected with BAC DNA for viral RNA synthesis and protein expression plasmids for N, P, L, and M2-1, together with pCAGGS-T7 polymerase using Lipofectamine LTX (Thermo Fisher Scientific, Waltham, MA, USA, Cat. No. 15338100). At 5–7 days post-transfection, the recovered viruses were grown in HEp-2 cells.

### 2.4. Conventional RT-PCR for MARCH8 mRNA

Conventional RT-PCR was performed to detect MARCH8 mRNA expression in A549 and MARCH8 knockdown A549 cells. Total cellular RNA was extracted using the MagExtractor-RNA- (Toyobo, Osaka, Japan, Cat. No. NPK-201F) according to the manufacturer’s protocol. cDNA was synthesized using the PrimeScript™ High Fidelity RT-PCR Kit (TaKaRa, San Jose, CA, USA, Cat. No. R022A) using oligo-dT primers. MARCH8 gene-specific primers were used for PCR amplification of MARCH8 cDNA. Primer sequences were designed according to previously reported methods [21] and are as follows:

Forward: 5′-TGCATCAGATCTCTGCCATT-3′
Reverse: 5′-TGGGACGTCATCTGCAACTTC-3′

β-actin mRNA was also amplified by PCR as an internal control to normalize variations in RNA quantity and quality across samples. The β-actin gene-specific primer set used was:

Forward: 5′-ACCAACTGGGACGACATGGAGAAA-3′
Reverse: 5′-TAGCACAGCCTGGATAGCAACGTA-3′

### 2.5. Immunofluorescence Assay

293T and HEp-2 cells were seeded into six-well plates (1 × 10^6^ cells/well) and transfected with protein expression plasmids using Lipofectamine LTX (Thermo Fisher Scientific, Cat. No. 15338100) or TransIT-LT1 reagent (Mirus Bio, Madison, WI, USA, Cat. No. MIR2300). In the viral infection experiment, at 24 h post-transfection (hpt), the transfected cells were infected with wt-RSV at a multiplicity of infection (MOI) of 1 for 72 h. At 48 or 96 hpt, the cells were fixed with 10% buffered formalin solution and permeabilized with 0.5% Triton-X 100. After blocking with Blocking One (Nakarai Tesque, Kyoto, Japan, Cat. 03953-95), the cells were incubated with primary antibodies (Abs) and subsequently with secondary antibodies conjugated to Alexa Fluor 488, 594, and 647 (Molecular Probes, Eugene, OR, USA). The following primary antibodies were used: rabbit anti-MARCH8 polyclonal antibody (pAb) (Proteintech, San Diego, CA, USA, Cat. No.14119-1-AP), rabbit anti-SH pAb raised against the cytoplasmic region of the SH protein (HNKTFELPRARVNT), and mouse anti-HA tag monoclonal antibody (mAb) (Proteintech, Cat. No. 66006-2-IG). Nuclear staining was carried out with 4′,6-diamidino-2-phenylindole (DAPI). Cells were observed under a fluorescence microscope (BZ-X810; Keyence, Osaka, Japan) and a laser scanning confocal microscope (FV3000, Olympus, Tokyo, Japan). The RGB plot profile plug-in in the ImageJ 1.53 software was used to generate fluorescence intensity plot profiles.

### 2.6. Viral Growth Kinetics

HEp-2 cells were transfected with the MARCH8 expression plasmid and centrifuged at 250× *g* for 5 min to improve the transfection efficiency. Under these conditions, we confirmed that the transfection efficiency of the mStrawberry expression plasmid exceeded 80%. Transfected HEp-2 cells and MARCH8 knockdown A549 cells were infected with wt-RSV and SH-K13R mutant viruses at MOI of 1.0. The culture medium was harvested at various time points, and viral titers were measured as the 50% tissue culture infectious dose (TCID_50_).

### 2.7. Western Blotting and Co-Immunoprecipitation

Transfected and/or infected cells were resuspended in cold RIPA lysis buffer (Santa Cruz Biotechnology, Dallas, TX, USA, Cat. No. sc-24948A) and centrifuged to remove the cell debris. The supernatants were then subjected to western blotting with rabbit anti-SH pAb and rabbit anti-MARCH8 pAb (Proteintech, Cat. No. 14119-1-AP), rabbit anti-HA tag pAb (MBL, Woods Hole, MA, USA, Cat. No. 561), rabbit anti-DYKDDDDK tag pAb (Proteintech, Cat. No. 20543-1-AP), mouse anti-F mAb (Abcam, Cambridge, MA, USA, Cat. No. ab24011), mouse anti-G mAb (Abcam, Cat. No. ab94966), and goat anti-RSV pAb (Acris, London, UK, Cat. No. BP1054). For drug treatment, 293T cells were co-transfected with the SH and MARCH8 expression plasmids. At 24 hpt, the cells were treated with 2.5 μM MG132 (Fuji film, Minato City, Japan, Cat. No. 139-18451) and 35 μM chloroquine (Sigma-Aldrich, St. Louis, MO, USA, Cat. No. C6628) 16 h before harvesting. For co-immunoprecipitation, the supernatants were mixed with rabbit anti-HA tag pAb and incubated at 4 °C for 60 min. Supernatants were mixed with protein A/G agarose (Santa Cruz Biotechnology, Cat. No. sc-2003) and incubated at 4 °C for 60 min. Immunoprecipitates were eluted from the agarose beads by boiling them with a SDS sample buffer and analyzed using western blotting.

### 2.8. TUNEL Assay

HEp-2 and MARCH8 knockdown A549 cells were used for TUNEL assay. HEp-2 cells were transfected with the MARCH8 expression plasmid. The cells were infected with wt-RSV and SH-K13R mutant viruses at an MOI of 1. At 5 dpi for HEp-2 cells or 8 dpi for MARCH8 knockdown A549 cells, the apoptotic infected cells were stained with Cell Meter Live Cell TUNEL Apoptosis Assay Kit Red Fluorescence (AAT Bioquest, Pleasanton, CA, USA, Cat. No. 22844), and then the apoptotic rate was calculated as the ratio of the number of apoptotic cells to the total number of cells using the Analyze Particles plug-in in ImageJ software.

### 2.9. Protein Structure Prediction

Docking of MARCH8 and SH proteins was performed using AlphaFold 3. The interaction interface between MARCH8 and the SH protein was modeled using the AlphaFold 3 Webserver [22]. The full-length sequences of MARCH8 (Sequence ID: NP_001002266.1) and SH (Sequence ID: AAG28084.1) were retrieved from the NCBI database and uploaded for modeling. The top-ranked model was visualized using PyMOL3.1.

### 2.10. Alignments for SH

To analyze similarities, the amino acid sequences of the SH protein were downloaded from GenBank (accessed on 26 July 2023). Strains with an uncertain sequence, unclear year of collection, or unclear area were excluded. Amino acid sequences from 180 strains extracted from GenBank that were 100% identical were removed from the data set. Finally, 60 RSV-A strains were collected, and their SH amino acid sequences were aligned. Sequence logos were drawn using Geneious Prime 2024.0.7 (https://www.geneious.com) (accessed on 18 October 2024).

### 2.11. Statistical Analysis

Data are presented as mean values with standard deviations from three independent experiments. Statistical significance was determined using Student’s *t*-test. *p* values < 0.05 or 0.01 were considered statistically significant.

## 3. Results

### 3.1. MARCH Family Proteins Target RSV-SH

**Figure 1:**
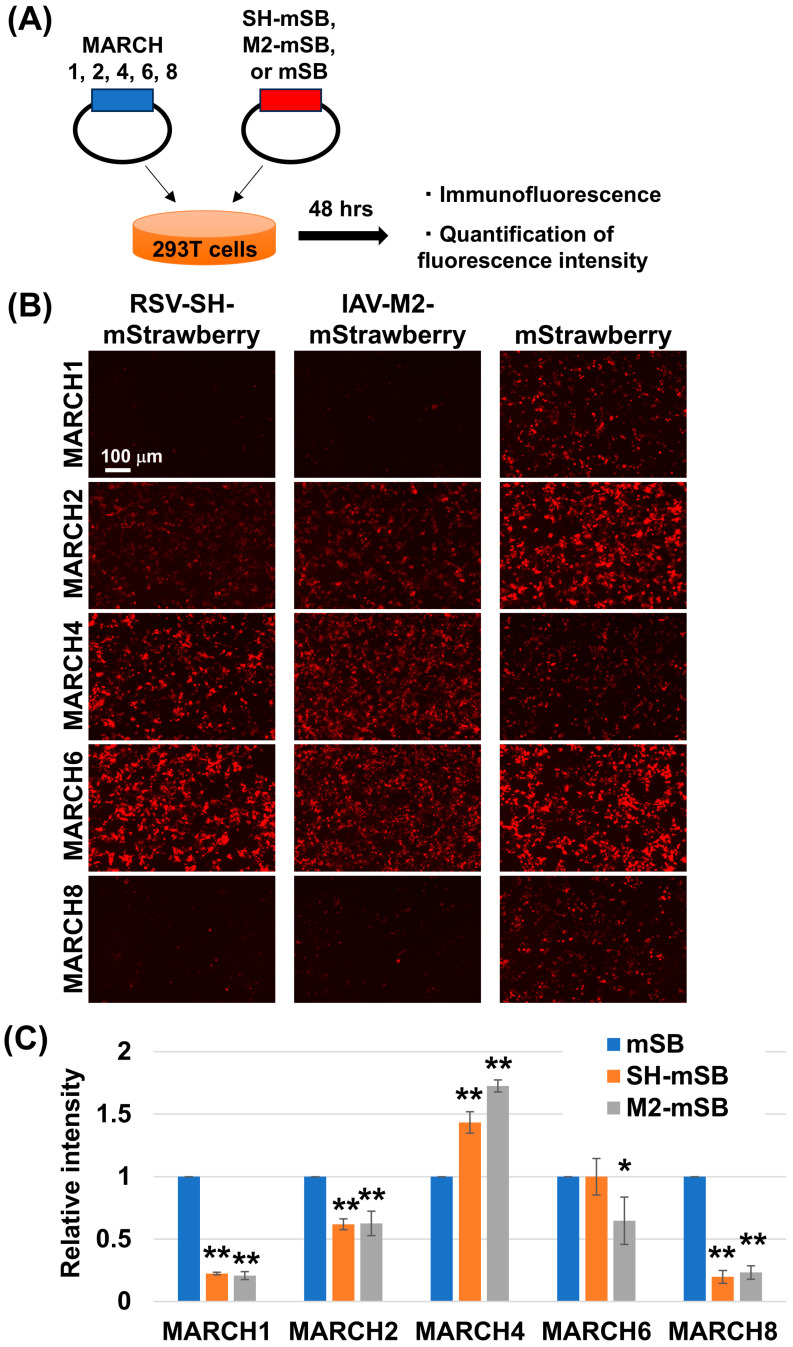
Reduction of RSV-SH and IAV-M2 by overexpression of MARCH1 and MARCH8. Schematic representation of the flow for transfection experiments using MARCH and RSV-SH fused with mStrawberry (mSB), influenza A virus (IAV) M2 fused with mSB, or mSB expression plasmids (**A**). 293T cells were co-transfected with MARCH1, 2, 4, 6, and 8 expression plasmids along with expression plasmids of RSV-SH-mSB, IAV-M2-mSB, and mSB. At 48 hpt, the cells were observed with fluorescence microscopy. All images were taken at the same magnification and exposure time (**B**). The fluorescence intensity acquired from at least five of each image was quantified using ImageJ. The relative fluorescence intensity of SH-mSB or M2-mSB to that of mSB is shown in the graph. Asterisks represent statistically significant differences using the Student’s *t*-test (**: *p* < 0.01, *: *p* < 0.05) (**C**).

Previous studies have demonstrated that the MARCH family proteins target the M2 protein of influenza viruses, leading to lysosomal degradation and inhibition of viral replication [19]. Given that M2 is a pentameric membrane protein structurally and functionally similar to the SH protein of RSV, we hypothesized that RSV-SH may also be a target of MARCH proteins. To test this hypothesis, we screened representative MARCH proteins for their ability to promote SH degradation (Figure 1A). Co-expression experiments revealed significant down-regulation of SH in cells expressing MARCH1 and MARCH8, whereas the expression of MARCH4 resulted in a slight increase in the SH level compared to that of mStrawberry as a control. (Figure 1B,C). RSV primarily infects epithelial cells in the bronchi and alveoli, where MARCH8 is predominantly expressed. Furthermore, MARCH8 has previously been reported to function to inhibit several viral replications [14,15,16,17,18,19]. Therefore, we focused our subsequent investigations on MARCH8.

### 3.2. MARCH8 Downregulates RSV-SH via the Ubiquitin-Lysosome Pathway

**Figure 2:**
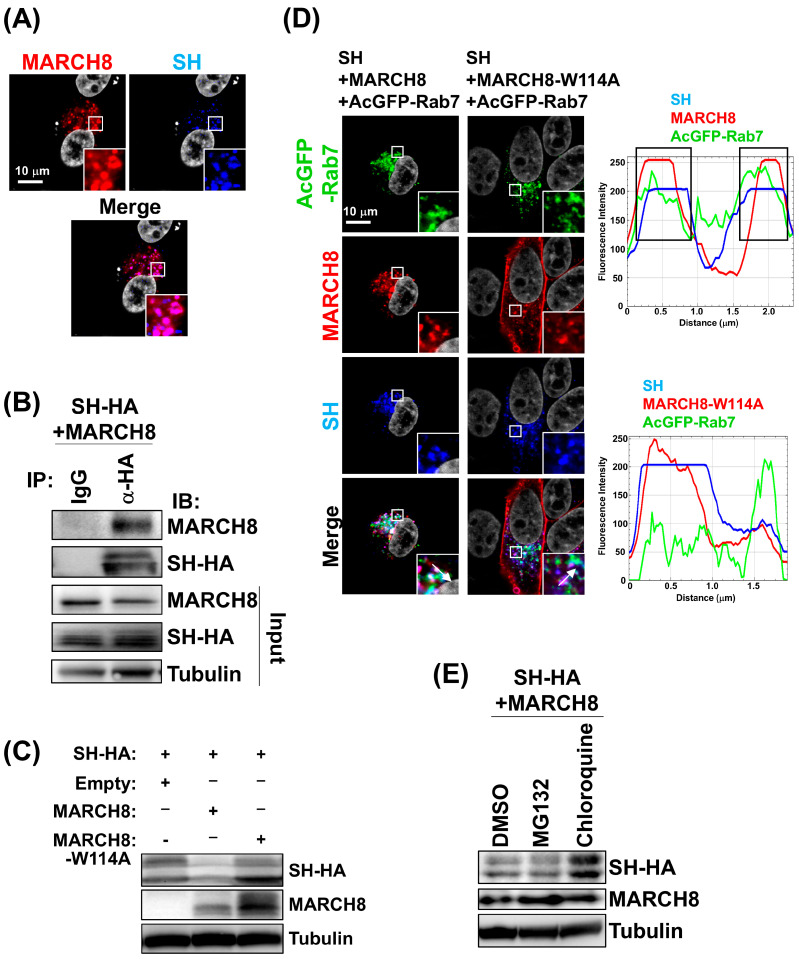
Lysosomal degradation of RSV-SH ubiquitinated by MARCH8. (**A**) HEp-2 cells were transfected with MARCH8-HA expression plasmid. After 24 h, the cells were then infected with wt-RSV at MOI of 1 for 72 h. Squares indicate co-localization areas. (**B**) For the co-immunoprecipitation assay, 293T cells expressing SH-HA and MARCH8 were incubated with anti-HA antibody or control IgG followed by the subsequent addition of Protein A/G agarose. (**C**) 293T cells were co-transfected with RSV-SH-HA and wt-MARCH8, and MARCH8-W114A mutant expression plasmids. At 48 hpt, the cells were subjected to western blotting using anti-HA or MARCH8 antibodies. (**D**) HEp-2 cells were co-transfected with expression plasmids for a combination of wt-SH plus MARCH8, and wt-SH plus MARCH8-W114A mutant along with late endosome/lysosome marker AcGFP-Rab7. Nuclei were stained with DAPI. Insets represent an enlargement of the areas indicated by a small square. Fluorescence profiles are shown with fluorescence intensity (y-axis) and distance (x-axis) according to the direction of the arrows within the enlarged squares. (**E**) 293T cells were co-transfected with SH and MARCH8 expression plasmids. At 24 hpt, the cells were untreated (DMSO control) or treated with MG132 or chloroquine for 16 h before harvest.

To elucidate the mechanism of MARCH8-mediated RSV-SH degradation, we first investigated the interaction between MARCH8 and SH using RSV-infected cells using immunofluorescence assay and co-immunoprecipitation analysis. Our results demonstrated that SH and MARCH8 co-localized in the cytoplasm in the virus-infected cells (Figure 2A) and SH co-immunoprecipitated with MARCH8 (Figure 2B). We then generated a W114A mutant of MARCH8, which is unable to bind E2 enzymes and lacks the E3 ligase activity. While wild-type MARCH8 effectively degraded RSV-SH, the W114A mutant did not (Figure 2C), indicating that the E3 ligase activity of MARCH8 is crucial for RSV-SH degradation.

To further investigate the degradation pathway, we conducted an immunofluorescence analysis using Rab7 as a late endosomal/lysosomal marker. In cells co-expressing SH and MARCH8, both proteins co-localized with Rab7-positive puncta, which coincided with reduced SH expression (Figure 2D). However, in cells co-expressing RSV-SH and MARCH8-W114A mutant, the protein was localized in the perinuclear region and plasma membrane, with minimal co-localization in Rab7-positive compartments. This observation is consistent with the inability to ubiquitinate substrate proteins for subsequent endocytosis, resulting in the accumulation of MARCH8-W114A at the plasma membrane. The fluorescence intensity profiles of SH, MARCH8, and Rab7 confirmed that these peaks were often correlated, but less correlation was observed between the fluorescence peaks of SH, MARCH8-W114A, and Rab7 (Figure 2D). To determine the fate of the ubiquitinated RSV-SH, we treated SH and MARCH8 co-transfected cells with either the proteasome inhibitor MG132 or the lysosomal inhibitor chloroquine. Although MG132 treatment did not affect SH degradation, chloroquine treatment significantly restored SH expression (Figure 2E).

These results strongly suggest that the MARCH8-mediated ubiquitination of RSV-SH leads to its lysosomal degradation.

### 3.3. MARCH8 Ubiquitinates RSV-SH on Lysine 13

**Figure 3:**
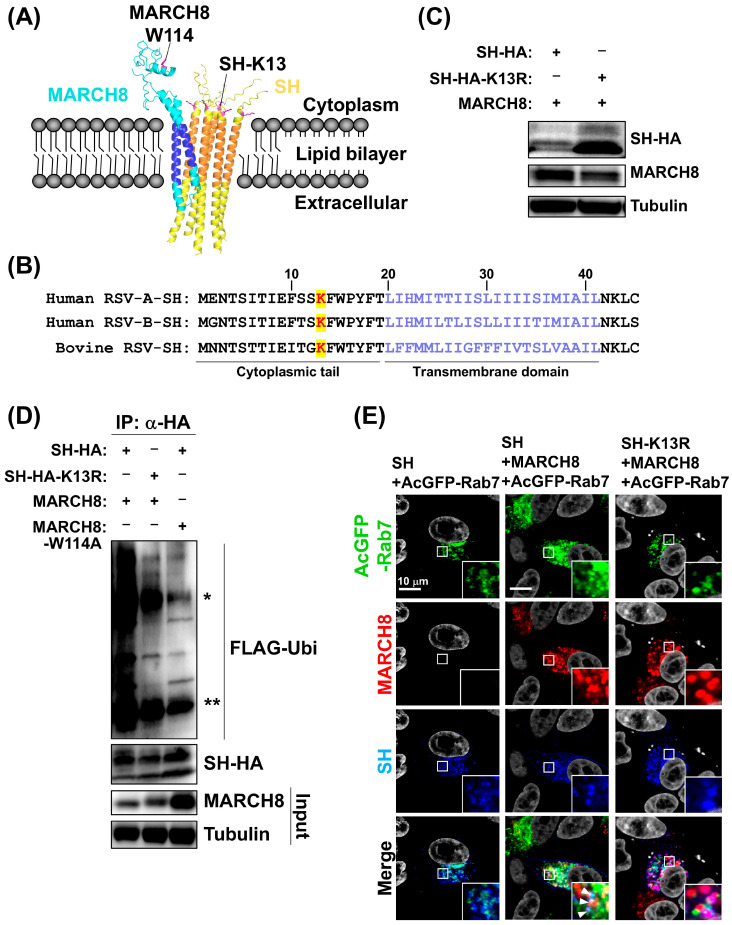
MARCH8 ubiquitinates RSV-SH on Lysine 13. (**A**) The complex structure of pentamer RSV-SH (yellow) and partial MARCH8 (cyan) is predicted using Alphafold 3 software. Transmembrane domains of MARCH8 and SH predicted by SOSUI system were highlighted in blue and orange colors, respectively. Tryptophan at amino acid position 114 (W114) in MARCH8 is the E2 enzyme binding site, and RSV-K13, which MARCH8 ubiquitinates, was predicted to be present together on the cytoplasmic side. (**B**) Amino acid alignment of human RSV-SH subgroup A and B and bovine RSV-SH. K13 residues in the cytoplasmic tail of SH and the transmembrane domain predicted by SOSUI are shown in red and purple, respectively. (**C**) 293T cells were co-transfected with wt-SH and SH-K13R mutant together with MARCH8 expression plasmids. (**D**) For ubiquitination of SH by MARCH8, 293T cells were co-transfected with wt-SH and wt-MARCH8, SH-K13R mutant and wt-MARCH8, and wt-SH and MARCH8-W114A mutant along with FLAG-tagged ubiquitin (FLAG-Ubi). Ubiquitination of SH was detected by western blotting using anti-FLAG antibody. * heavy chain, ** light chain. (**E**) HEp-2 cells were co-transfected with expression plasmids of wt-SH and a combination of wt-SH plus MARCH8, and wt-SH plus MARCH8-W114A mutant along with AcGFP-Rab7. Insets represent an enlargement of the areas indicated by a small square and arrowheads show the co-localization area of SH and Rab7.

Next, we sought to identify the ubiquitination sites of RSV-SH. Initially, we predicted the joint structure of RSV-SH and MARCH8 protein complexes using AlphaFold 3. The predicted biomolecular structures revealed that RSV-SH and MARCH8 interacted via their respective transmembrane domains, with the RING domain of MARCH8 in close proximity to the N-terminal region of pentameric RSV-SH on the cytoplasmic side (Figure 3A). A detailed examination of the cytoplasmic side of RSV-SH revealed that the lysine residue at position 13 of SH protein (K13) was the only lysine residue present in this region (Figure 3A). Amino acid alignment analysis showed that this residue was conserved in RSV-A, RSV-B, and bovine RSVs (Figure 3B). In addition, analysis of over 100 RSV clinical isolates confirmed that K13 in the SH was highly conserved among RSV strains (Supplementary Figure S1).

To test whether K13 was the target of MARCH8-mediated ubiquitination, we constructed an SH-K13R mutant in which lysine was substituted for arginine. Co-expression studies demonstrated that while wild-type SH was degraded by MARCH8, the SH-K13R mutant was resistant to degradation (Figure 3C). Consistently, immunoprecipitation-based ubiquitination assay showed that wild-type SH, but not the SH-K13R mutant, underwent poly-ubiquitination by MARCH8 (Figure 3D). In addition, it was also shown that the MARCH8-W114A mutant did not ubiquitinate wild-type SH (Figure 3D). Immunofluorescence analysis further demonstrated that wild-type SH localized to Rab7-positive perinuclear puncta with MARCH8, whereas the SH-K13R mutant did not co-localize with Rab7 even with MARCH8 overexpression (Figure 3E).

Collectively, these results indicate that K13 is the primary ubiquitination site of RSV-SH targeted by MARCH8. Our current results also indicate that RSV with SH-K13 mutation is able to escape MARCH8 suppression.

### 3.4. Degradation of SH by MARCH8 Promotes Apoptosis of Infected Cells and Inhibits Virus Replication

**Figure 4:**
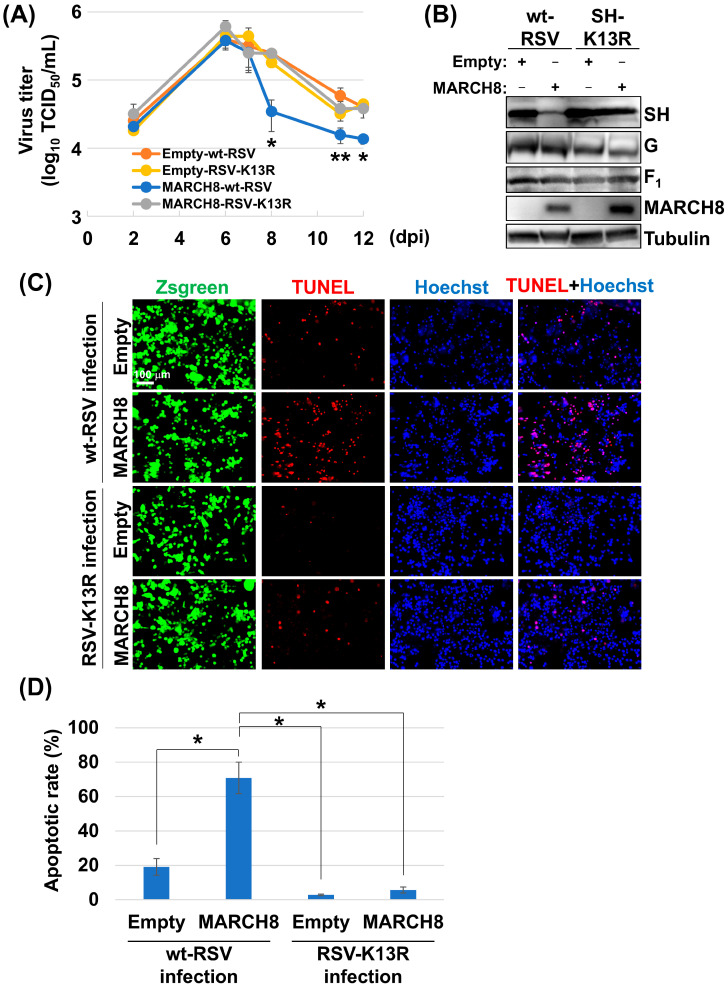
MARCH8-mediated ubiquitination of RSV-SH promotes apoptosis and inhibits virus persistence. HEp-2 cells were transfected with the MARCH8 expression plasmid or an empty plasmid. The cells were infected with the wt-RSV or RSV-K13R mutant virus. At various time points (indicated), aliquots of the cell culture medium were collected, and the viral titer in the medium was measured as TCID_50_. Each day after infection, viral titers in transfected cells were compared with viral titers in samples transfected with empty vectors and infected with wt-RSV. Asterisks indicate statistically significant differences using the Student’s *t*-test (* *p* < 0.05, ** *p* < 0.01) (**A**). HEp-2 cells were transfected and infected with wt-RSV or RSV-K13R viruses, as described in Figure 4A (**B**). HEp-2 cells were transfected and infected with wt-RSV or RSV-K13R mutant viruses. The cells were subjected to a TUNEL assay to detect cell death by apoptosis (**C**). The apoptosis rate was calculated as the ratio of apoptotic cells to the total number of cells using the Analyze Particles plug-in of the ImageJ software. Asterisks indicate statistically significant differences using the Student’s *t*-test (* *p* < 0.01) (**D**).

Given previous reports that RSV-SH inhibits cell apoptosis by suppressing TNF-alpha signaling [12], we investigated whether MARCH8-mediated SH degradation affects cellular apoptosis and viral replication. We transfected HEp-2 cells with MARCH8 and subsequently infected them with either the wild-type RSV or the SH-K13R mutant virus at a multiplicity of infection (MOI) of 1. In wild-type RSV-infected cells, MARCH8 overexpression resulted in a significant decline in viral titers at 8 dpi. Conversely, in SH-K13R mutant virus-infected cells, viral titers remained relatively constant despite MARCH8 overexpression (Figure 4A). Immunoblotting analysis at 7 dpi showed that MARCH8 overexpression specifically reduced SH expression in wild-type RSV-infected cells, whereas other viral membrane proteins (F and G) remained unaffected. This reduction was not observed in SH-K13R mutant virus-infected cells (Figure 4B). TUNEL assays demonstrated a significantly higher percentage of apoptotic cells in MARCH8-overexpressing wild-type RSV-infected cells than in SH-K13R mutant virus-infected or control cells (Figure 4C,D).

**Figure 5:**
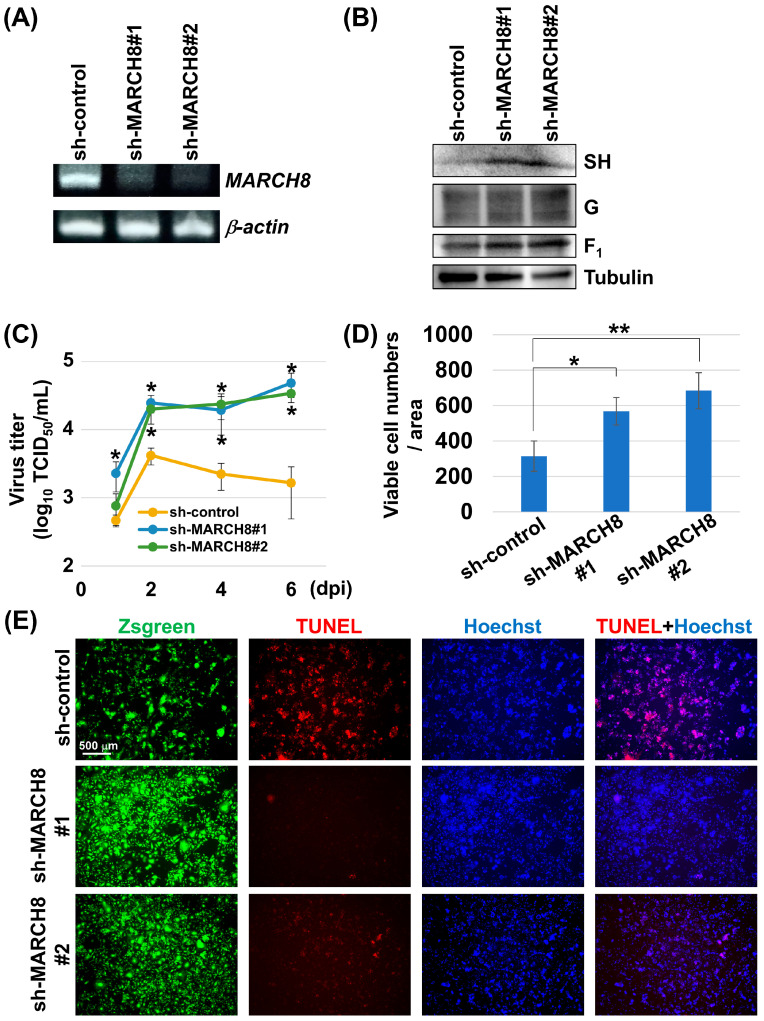
MARCH8 knockdown inhibits virus-induced apoptosis and enhances viral replication. MARCH8 mRNA expression levels in control (sh-control) and MARCH8-knockdown (KD) (sh-MARCH8#1 and sh-MARCH8#2) A549 cell lines were examined by conventional RT-PCR using primers specific for MARCH8 (**A**). Control and MARCH8-KD A549 cells were infected with wt-RSVand the cells were collected and subjected to western blotting with the appropriate antibodies to detect each protein (**B**). Control and MARCH8-KD A549 cells were infected with wt-RSV. At various time points (indicated), aliquots of the cell culture medium were collected, and the viral titer was measured as TCID_50_ (**C**). The virus-infected cells were fixed and stained with Hoechst stain, and the number of viable cells from more than 10 images was counted (**D**). The TUNEL assay was performed to quantify cell death by apoptosis. Representative images in TUNEL assays of control and MARCH8-KD cells (**E**). Viral titers and the number of viable MARCH8-KD cells were compared with those in control cells. Asterisks indicate statistically significant differences using the Student’s *t*-test (*: *p* < 0.05, **: *p* < 0.01).

To further validate these findings, we generated MARCH8 knockdown A549 lung epithelial cells using specific shRNAs. RT-PCR confirmed the reduced expression of MARCH8 mRNA in these cells (Figure 5A). Subsequent RSV infection experiments revealed that MARCH8 knockdown cells exhibited higher levels of SH protein (Figure 5B), increased viral proliferation (Figure 5C), higher cell survival rates (Figure 5D), and lower levels of apoptotic markers than control shRNA-expressing cells (Figure 5E).

Collectively, these results indicate that MARCH8 specifically targets SH in infected cells, promoting its degradation and consequently inducing apoptotic cell death in RSV-infected cells. These mechanisms appear to play a crucial role in regulating RSV replication and cell survival during infection.

## 4. Discussion

The innate immune system plays a crucial role in the initial defense against viral infections. Among the various components of this system, the Membrane-Associated RING-CH (MARCH) family of proteins has emerged as a significant player in the regulation of immune response. Among the MARCH family proteins, MARCH8 is a potent suppressor of the replication of a broad spectrum of viruses, and its functional role and detailed molecular mechanisms are being elucidated. Understanding the antiviral functions of MARCH8 not only provides insights into host–pathogen interactions but also opens new avenues for therapeutic interventions. In this study, we reveal a novel mechanism of MARCH8-mediated antiviral defense against RSV. We demonstrated that MARCH8 specifically targets the RSV-SH protein for degradation via the ubiquitin-lysosome pathway. This mechanism not only restricts viral replication but also influences virus-induced apoptosis, potentially limiting viral spread. Our study unveils a critical host–pathogen interaction by highlighting the role of MARCH8 in SH protein degradation and opens new avenues for exploring host-directed antiviral therapies.

Recent studies have revealed that the MARCH family proteins possess direct antiviral properties, extending their role beyond immune modulation [14,15,16,17,18,19]. The MARCH family proteins are E3 ubiquitin ligases that primarily function by modulating the surface expression of various immune-related proteins via ubiquitination-mediated degradation or trafficking alterations [23]. The MARCH family comprises 11 human members, each with distinct but often overlapping functions in immune regulation and cellular homeostasis [24]. In particular, MARCH8 is a potent antiviral factor against a range of viral species; the molecular basis of the antiviral function of MARCH8 generally lies in its ability to regulate protein trafficking and degradation. By targeting viral envelope proteins for ubiquitination, MARCH8 can alter their cellular localization and direct them toward proteasomal degradation, thereby preventing viral assembly and release [19]. MARCH8 inhibits HIV-1 infection by downregulating the viral envelope glycoprotein and reducing its incorporation into virions [17]. Additionally, MARCH8 has also been identified as a host restriction factor for SARS-CoV-2, where it inhibits viral replication through the degradation of the viral nucleocapsid protein [25]. The mechanisms by which MARCH proteins exert their antiviral effects are varied and often virus-specific. In some cases, they directly target viral proteins for degradation, while in others, they modulate host factors essential for viral replication [18,26]. For example, furin-mediated cleavage, which is crucial for viral glycoprotein activation, is inhibited by MARCH8, which further blocks the maturation and entry of avian Influenza Virus H5N1 [18]. It is important to note that the antiviral effects of MARCH proteins are not universal. Some viruses have evolved mechanisms to counteract or exploit MARCH proteins for their benefit. For instance, Kaposi’s sarcoma-associated herpesvirus (KSHV) encodes viral MARCH proteins that mimic host MARCH functions and potentially subvert the antiviral response [26]. The present results also show that RSVs harboring the K13 mutation in SH can escape from the MARCH8 suppression. Such virus–host antagonism may have implications for virus evolution and epidemics. On the other hand, RS viruses with a mutation in the K13 residues have not yet been found in the viral genome database. This may be because the conservation of this amino acid is essential for SH function. More detailed molecular and genetic analyses will be needed to answer these intriguing questions.

The intricate interplay between viral infection and host cell apoptosis is a critical aspect of viral pathogenesis and host defense mechanisms [27]. Although apoptosis of infected cells can serve as a host defense mechanism to limit viral spread, many viruses have evolved strategies to inhibit this process. For example, human papillomavirus (HPV) expresses the E6 protein, which targets the tumor suppressor p53 for degradation, thereby preventing apoptosis [28]. Similarly, the adenovirus E1B-19K protein functions as a Bcl-2 homolog, inhibiting pro-apoptotic proteins such as Bax and Bak [29]. Furthermore, the Epstein-Barr virus (EBV) produces BHRF1, another Bcl-2 mimic, which blocks the release of cytochrome c from the mitochondria [30]. These examples illustrate the diverse mechanisms used by viruses to prolong the survival of infected cells, thereby facilitating their replication and spread. Our findings suggest that the RSV-SH protein functions as an anti-apoptotic factor, potentially promoting viral persistence and spread within the host, consequently enhancing pathogenicity. Notably, our current study identified a novel host counteraction to this viral strategy, in which the host protein MARCH8 appeared to target the SH protein for ubiquitin-dependent degradation, effectively neutralizing its anti-apoptotic activity. This host-mediated degradation of the SH protein may facilitate the rapid elimination of virus-infected cells through apoptosis, thereby limiting viral replication and mitigating tissue damage.

RSV is the major pathogen of respiratory tract infections worldwide, often causing severe respiratory infections in infants and the elderly. The role of RSV-SH in virus pathogenicity and infectivity has not been fully understood. SH proteins can form pentameric ion channels in lipid bilayers [9,12]. Although this ion channel function was initially thought to be essential for viral replication, studies with RSV lacking the SH gene (RSVΔSH) have overturned this theory. Rather, evidence is accumulating to suggest that SH plays an important role in RSV pathogenicity and immune evasion, independent of its ion channel function. Studies using recombinant RSVΔSH have demonstrated attenuated viral growth in vivo and reduced lung pathology in animal models [10]. Indeed, SH can activate the NLRP3 inflammasome, leading to IL-1β production, which may contribute to RSV-induced inflammation [31]. These data suggest that SH plays an important role in the inflammatory response in RSV infection. Also, SH protein inhibits apoptosis of infected cells by blocking TNF-α signaling [12]. As TNF-α is a major inflammatory cytokine and is involved in inflammation and pathogenesis in infected hosts, MARCH8 may also be involved in the suppression of disease progression and severity via SH degradation. To clarify the anti-inflammatory function of MARCH8, further detailed in vivo analysis using animal models is needed.

Our findings demonstrate that MARCH8 acts as a potent restriction factor against RSV through selective degradation of the SH protein. These results not only advance our understanding of host–pathogen interactions but also raise intriguing possibilities for therapeutic applications. Currently, no specific small molecule modulators directly targeting MARCH8 have been identified, although several physiological factors are known to regulate its expression and activity. For instance, the anti-inflammatory cytokine IL-10 has been reported to upregulate MARCH8 expression in immune cells, particularly in monocytes and dendritic cells [32]. This regulation suggests that the activity of MARCH8 is integrated within broader immune regulatory networks. Moreover, MARCH8 represents a promising therapeutic target for several reasons. First, as an E3 ubiquitin ligase, it belongs to a protein family that has proven amenable to drug development, as exemplified by successful therapeutic targeting of other E3 ligases in various disease contexts [33,34]. Second, its broad-spectrum antiviral activity suggests that MARCH8-based therapeutics might be effective against multiple viral pathogens [18,19,35]. Third, its cellular localization and structure could potentially allow for small molecule intervention [35,36]. However, therapeutic manipulation of MARCH8 requires careful consideration of potential side effects. MARCH8 regulates multiple cellular proteins, including important immune receptors such as IL-1RAP and CD44, as well as trafficking proteins like VAMP3 and CD166 [37,38,39]. Enhanced MARCH8 activity could, therefore, impact various cellular processes, including immune responses and cellular trafficking pathways, resulting in the potential disruption of normal cell–cell communication. Future studies should focus on developing specific MARCH8 modulators while carefully evaluating their effects on cellular homeostasis. The development of tissue-specific or activation-dependent MARCH8 modulators might help minimize potential side effects while maintaining therapeutic efficacy. Additionally, a detailed investigation of the regulatory mechanisms of MARCH8 could reveal more subtle intervention points that allow for fine-tuned therapeutic approaches.

In conclusion, we have demonstrated that MARCH8 restricts RSV replication by degrading the viral SH protein and enhancing cellular apoptosis, which is a significant component of the host antiviral defense system. Their diverse mechanisms of action and the ability of MARCH family proteins to target multiple stages of the viral lifecycle make them promising subjects for further research in the field of antiviral immunity. As our understanding of these proteins increases, so does the potential for the development of novel antiviral therapies that exploit their functions.

## Data Availability

The data that support the findings of this study are available from the corresponding author upon reasonable request.

## Supplementary Materials

The following supporting information can be downloaded at: https://www.mdpi.com/article/10.3390/v16121935/s1, Figure S1: Alignment of human RSV subgroup A SH amino acid sequences. Figure S2: Photographs of the original gel from all western blot and RT-PCR.
