# Supplementary material for: MARCH8 Restricts RSV Replication by Promoting Cellular Apoptosis Through Ubiquitin-Mediated Proteolysis of Viral SH Protein
Source: Viruses. 2024 Dec 18;16(12):1935. doi: 10.3390/v16121935 (PMC11680241; doi:10.3390/v16121935)
Supplement: Supplementary file 1 [file viruses-16-01935-s001.zip › viruses-3344722-supplementary.pdf]

Supplementary information

MARCH8 restricts RSV replication by promoting cellular apoptosis through ubiquitin-mediated proteolysis of viral SH protein

Takashi Okura [1], Tatsuki Takahashi [2], Taichi Kameya [1,3], Fuminori Mizukoshi [1], Yusuke Nakai [1], Masatoshi Kakizaki [1], Mayuko Nishi [1], Noriyuki Otsuki [1], Hirokazu Kimura [4], Kei Miyakawa [5, 6], Kazuya Shirato [1], Wataru Kamitani [b], and Akihide Ryo [1, 6, *]

[1] Department of Virology 3, National Institute of Infectious Diseases, Musashimurayama, Tokyo, Japan.

[2] Department of Infectious Diseases and Host Defense, Gunma University Graduate School of Medicine.

[3] Life Science Laboratory, Technology and Development Division, Kanto Chemical Co., Inc., Kanagawa, Japan.

[4] Department of Health Science, Gunma Paz University Graduate School of Health Sciences, Gunma, Japan.

[5] Research Center for Influenza and Respiratory Viruses, National Institute of Infectious Diseases, Tokyo, Japan.

[6] Department of Microbiology, Graduate School of Medicine, Yokohama City University, Yokohama, Japan

* Correspondence: Akihide Ryo

Email: aryo@niid.go.jp

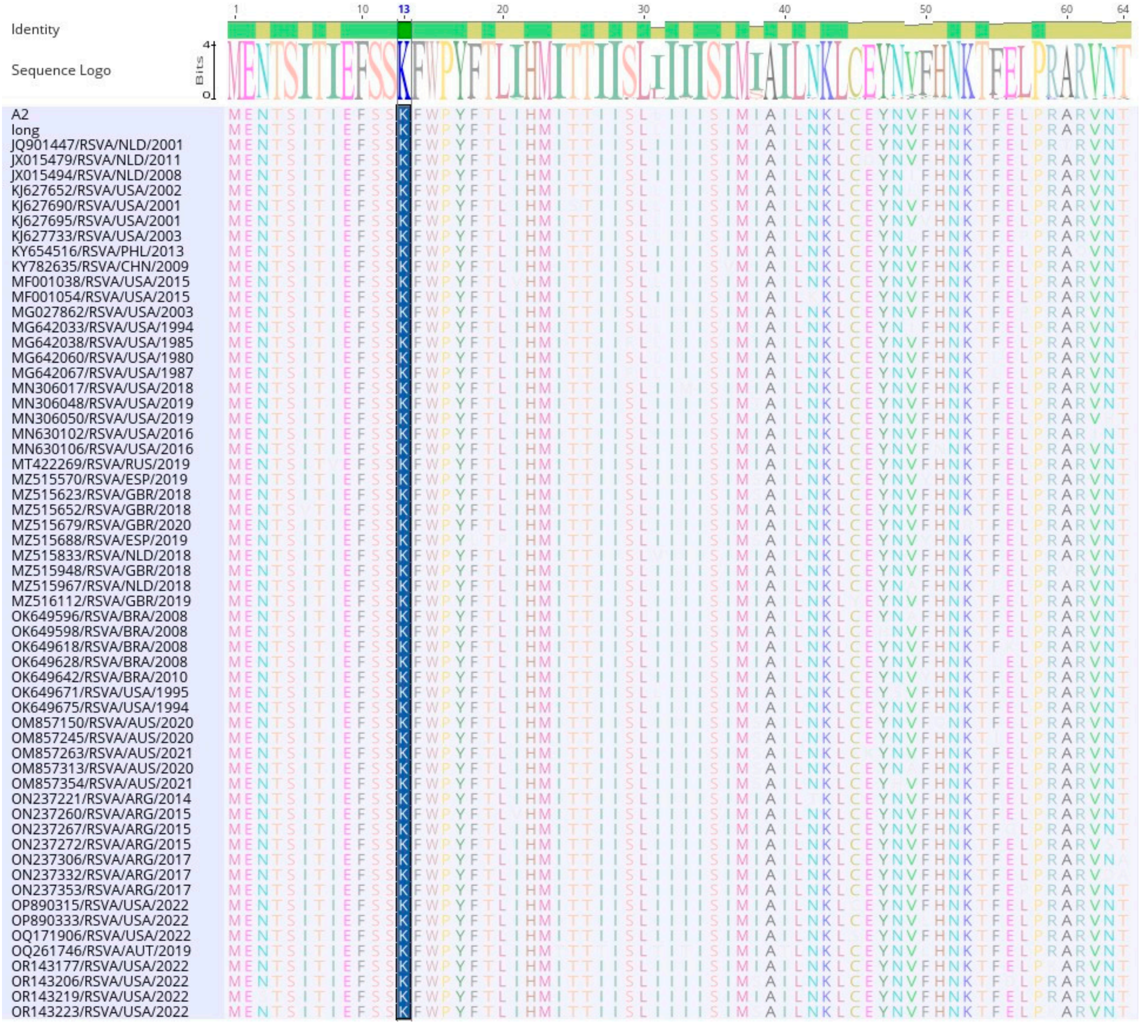

Supplementary Figure S1. Alignment of human RSV subgroup A SH amino acid sequences. Alignment of SH amino acid sequences from 60 strains of RSV-A including A2 and Long strains. Lysine 13 of SH, the critical ubiquitin acceptor site for MARCH8-mediated ubiquitination identified in this study, is highlighted.

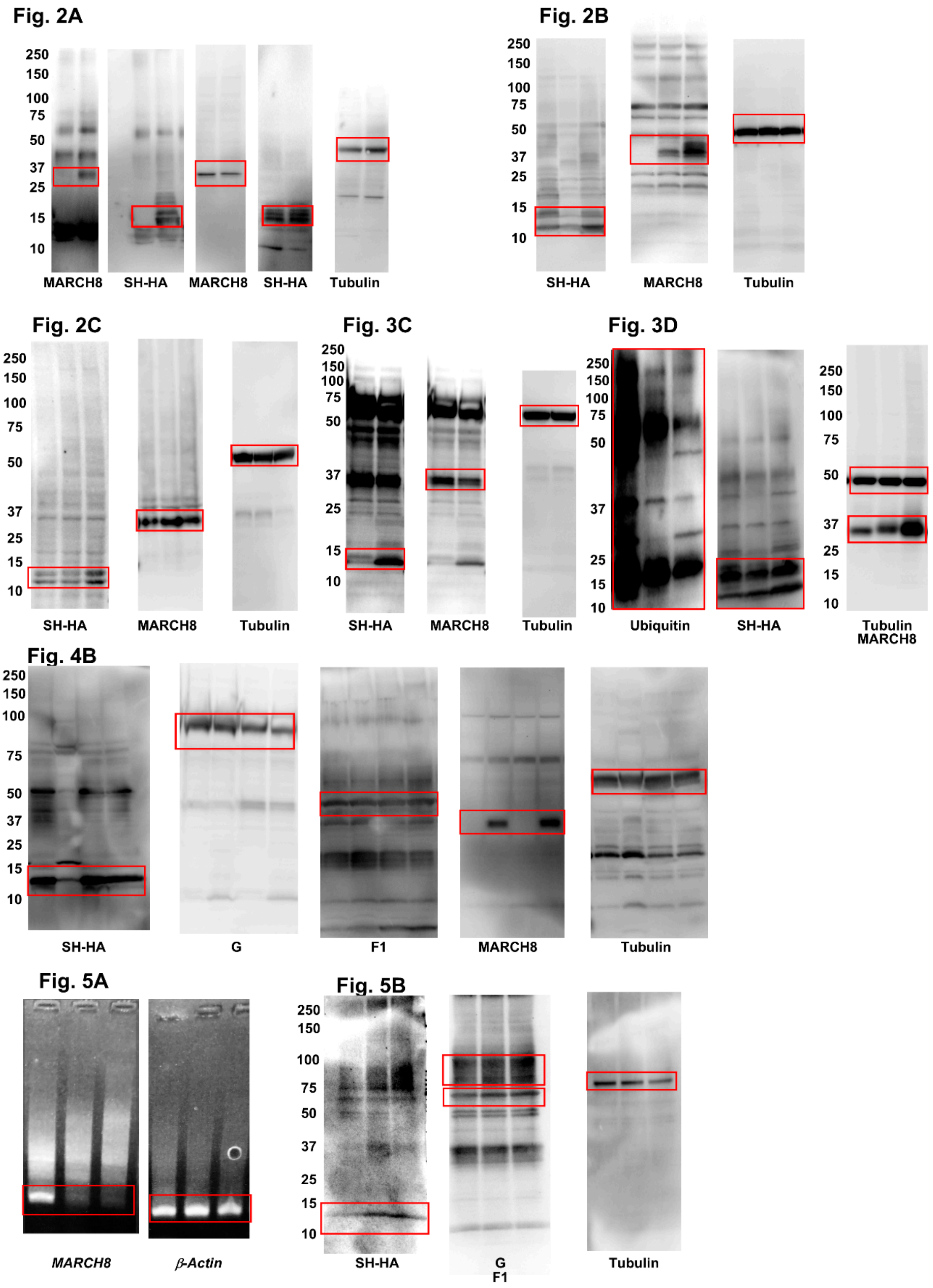

Supplementary Figure S2. Photographs of the original gel from all western blot and RT-PCR, respectively, used in this study are shown.
